# Anchoring group regulation in semiconductor/molecular complex hybrid photoelectrode for photoelectrochemical water splitting

**DOI:** 10.1002/smo.20240056

**Published:** 2024-12-08

**Authors:** Xiangyan Chen, Fujun Niu, Tongxiang Ma, Qingyu Li, Shaopeng Wang, Shaohua Shen

**Affiliations:** ^1^ Northwest Institute for Non‐ferrous Metal Research Xi'an Shaanxi China; ^2^ School of Advanced Energy Shenzhen Campus of Sun Yat‐sen University Shenzhen China; ^3^ State Key Laboratory of Multiphase Flow in Power Engineering International Research Center for Renewable Energy Xi'an Jiaotong University Xi'an Shaanxi China

**Keywords:** anchoring groups, hybrid systems, molecular catalysts, photoelectrochemical water splitting, semiconductor photoelectrodes

## Abstract

Rational interface engineering via regulating the anchoring groups between molecular catalysts and light‐absorbing semiconductors is essential and emergent to stabilize the semiconductor/molecular complex interaction and facilitate the photocarriers transport, thus realizing highly active and stable photoelectrochemical (PEC) water splitting. In this mini review, following a showcasing of the fundamental details of hybrid PEC systems containing semiconductor photoelectrodes and molecular catalysts for water splitting, the state‐of‐the‐art progress of anchoring group regulation at semiconductor/molecular complex interface for efficient and stable PEC water splitting, as well as its effect on charge transfer kinetics, are comprehensively reviewed. Finally, potential research directions aimed at building high‐efficiency hybrid PEC water splitting systems are summarized.

## Graphical abstract

Rational interfacial anchoring groups engineering between semiconductor photoelectrodes and molecular catalysts, including regulating the type and density of anchor, optimizing the length and conjugation property of linker, etc., is of great significance to stabilize the semiconductor/molecular complex interaction and facilitate charge carriers transport, thus realizing high‐efficiency and stable photoelectrochemical water splitting.

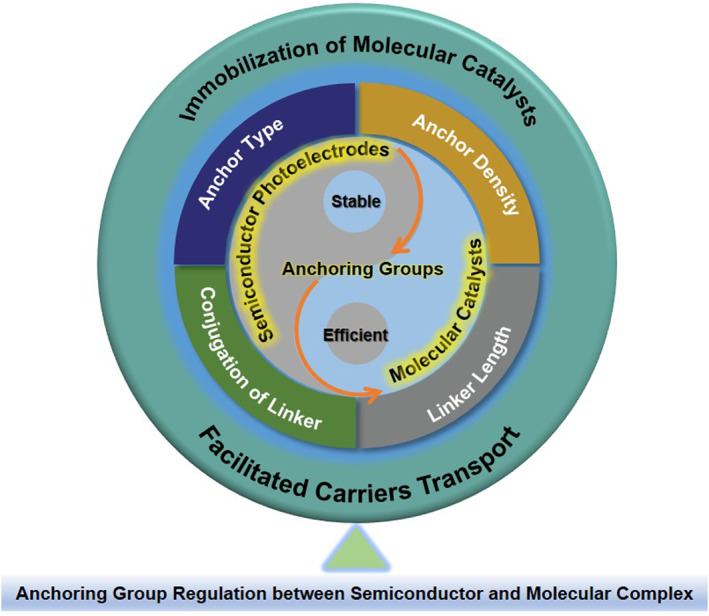

## 1 INTRODUCTION

Water splitting using photoelectrochemical (PEC) cells is a promising means to store solar energy in the form of fuels in an environmentally friendly and sustainable manner.[^1^, ^2^, ^3^] In a PEC cell, the key component is the photoelectrode (including photoanode and photocathode), which is responsible for solar energy harvesting, charge separation and transfer, and subsequent water oxidation/reduction reaction.[^4^, ^5^] It is intrinsically difficult to develop a single semiconductor photoelectrode simultaneously meeting the demands of suitable band structure for broadened sunlight absorption, efficient electron‐hole pairs separation and transport, and matched energy level for feasible water redox reactions. The past few decades have witnessed a rapid and full development of photoelectrode modification technologies, such as composite construction,[^6^, ^7^, ^8^] surface modification,[^9^, ^10^, ^11^] defect engineering,[^12^, ^13^, ^14^] etc., to address the above issues. Among these modification strategies, coupling light‐absorbing semiconductors with molecular catalysts to form hybrid PEC systems combines the merits of well‐defined molecular active sites with the broad optical absorption of semiconducting materials, showing promising potentials in obtaining high‐efficiency PEC water splitting performances.[^15^, ^16^, ^17^, ^18^, ^19^] For example, Sun et al. constructed a hybrid PEC system, in which the molecular catalyst of Co_4_O_4_ cubane was adsorbed on a BiVO_4_ photoanode, producing a photocurrent density of 5 mA cm^−2^ at 1.23 V versus reversible hydrogen electrode (RHE) and a high solar‐energy conversion efficiency of 1.84%.^[20]^ Recently, Wang et al. demonstrated that a significantly improved efficiency and stability of p‐Si photocathode for H_2_ production was achieved by anchoring the N_5_‐chelated nickel and cobalt catalyst (M(N_5_)^HA^, M = Ni, Co) on p‐Si surface.^[21]^

Although greatly boosted PEC water splitting performances have been obtained for semiconductor photoelectrodes with surface modified by molecular catalysts, there is still a gulf to be crossed between the reported hybrid PEC systems and their wide applications due to the difficulty of maintaining high‐efficiency performance within long‐term PEC water splitting operation.[^22^, ^23^] Until now there have been a number of various strategies reported for optimization of the hybrid semiconductor/molecular complex PEC system, mainly focusing on structural design of molecular catalyst,[^24^, ^25^, ^26^] interface modulation between molecular complex and semiconductor,[^27^, ^28^, ^29^] coupling with other modification methods,[^30^, ^31^, ^32^] electrolyte engineering,[^33^, ^34^] etc., to realize highly efficient and stable PEC water splitting performances. It has been validated that a rational interface engineering between semiconductor and its surface‐attached molecular catalysts plays a pivotal role in preventing molecular complexes detachment from semiconductor surfaces, significantly increasing the hybrid system stability. The interfacial interaction modes between semiconductors and molecular complexes have been mainly covered with noncovalent interactions (e.g. adsorption,[^23^, ^35^] collision,^[36]^ and π‐π stacking^[37]^) and covalent linkage.[^38^, ^39^, ^40^] Among the noncovalent interactions, direct adsorption can be achieved by casting a molecular catalyst solution on a photoelectrode with subsequent drying to acquire the hybrid system, and collision occurs at the interface between freely diffusing molecules and electrodes. The π‐π stacking, can be obtained by connecting molecular complex onto semiconductor surface via carbon material interlayers or polyaromatic sites. Compared to noncovalent interactions, covalent linkages could build chemical bonds between molecular complexes and semiconductors. Therefore, the order of collision < adsorption < π‐π stacking < covalent linkage should be followed in terms of interaction strength. Moreover, the covalent linkage is also the best choice for efficient carrier transport from semiconductor electrode to molecular catalyst via building a charge transfer channel. It has been highly emphasized that the type and density of anchoring group, covalently linking semiconductor and molecular complex, could influence the catalytic activity and stability of the hybrid photoelectrode for PEC water splitting. In addition, the length and conjugation property of the linker between the molecular catalyst and anchor could also make an impact on interfacial charge transfer kinetics, thus affecting the PEC reactivity. Even though there have been a few review papers relating to the topic of molecular catalyst ‐ modified semiconductor photoelectrodes for PEC water splitting,[^41^, ^42^, ^43^] the important point of strengthening the hybrid PEC device performance via performing the semiconductor/molecular complex interfacial engineering has not always received full attention in this field, and a detailed investigation on semiconductor/molecular complex interfacial reaction mode regulation for enhanced PEC performance is still lacking.

In this mini review, starting with a general overview of the hybrid PEC systems with light‐absorbing semiconductor photoelectrodes surface attached by molecular catalysts for water redox reactions, the most recent advancements of anchoring group regulation at semiconductor/molecular complex interface for efficient and stable PEC water splitting, as well as its impact on charge transfer kinetics, are comprehensively reviewed. Finally, the design principles for optimization of the interfacial bonding mode and then fabricating high‐efficiency hybrid PEC systems are proposed. It is highly believed that this mini review timely offers specific principles from the perspective of interface design for building efficient and stable semiconductor/molecular complex hybrid PEC systems.

## 2 SYSTEM OF LIGHT‐ABSORBING SEMICONDUCTOR WITH MOLECULAR CATALYST ATTACHED TO SURFACE

**FIGURE 1:**
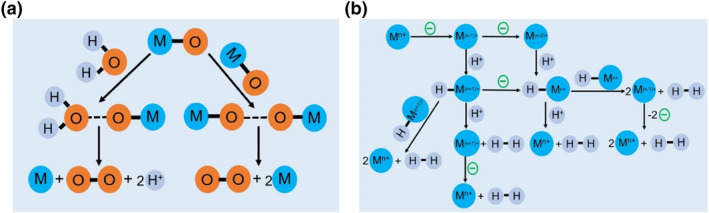
Schematic diagrams of (a) two mechanistic pathways to form an O‐O bond for molecular catalyst and (b) proposed mechanistic pathways for H_2_ generation at a metal center of M^n+^.

To the best of our knowledge, the PEC water splitting process for a light‐absorbing semiconductor photoelectrode with surface modified by molecular complex as catalyst involves three steps, that is, (i) light absorption exited electron‐hole pairs, (ii) photocarrier separation and transport to molecular catalyst, and (iii) water oxidation or reduction reaction at molecular catalyst following a specified reaction pathway. For water oxidation, the O‐O forming step could be summarized as interaction between two M‐O groups (I2M) and nucleophilic attack by water (WNA) (Figure 1a).^[44]^ The I2M can take place by reductive coupling and reductive elimination or by radical coupling in an intra‐ or inter‐molecular manner, while the WNA happens when a water molecule attacks the oxo group from the M‐O moiety which is sufficiently electrophilic. As for water reduction, the photoelectrons generated in semiconductor transfer to molecular catalysts and then reduce water to hydrogen based on the metal center (M^n+^) in the molecular complex. Typically, reduction of M^n+^ followed by protonation affords the key intermediate hydride H‐Mn^+^ that could generate H_2_ by a monometallic pathway or with a second hydride to give H_2_ by a bimetallic pathway (Figure 1b).

In hybrid PEC water splitting cells, numerous semiconductor photoelectrodes, including TiO_2_, WO_3_, Fe_2_O_3_, BiVO_4_, etc. for water oxidation, and Si, GaP, InP, Cu_2_O, etc. for water reduction, have been extensively developed.[^45^, ^46^, ^47^, ^48^, ^49^, ^50^, ^51^] In addition, Co‐based, Ir‐based, Ru‐based, NiFe‐based and Mo‐based molecular complexes have been widely adopted as water oxidation catalysts (WOCs) for O_2_ evolution,[^26^, ^36^, ^40^, ^52^, ^53^, ^54^] while the Fe‐based, Co‐based, Ni‐based, and Cu‐based molecular water reduction catalysts (WECs) have been deeply studied for H_2_ evolution.[^55^, ^56^, ^57^, ^58^] These metal coordinated molecular complexes, with merits of tunable physicochemical properties, high atom utilization, and ease in disclosing reaction mechanisms,^[18]^ could modify semiconductor surface and accelerate charge transfer at the electrode/electrolyte interface, thus boosting the PEC water splitting performances.[^23^, ^59^] For instance, Chen et al. reported a hybrid PEC system consisting of Cu_2_O as the light absorber and [Co(tpy)(phen)Cl]Cl as the WEC.^[51]^ The obtained Cu_2_O/[Co(tpy)(phen)Cl]Cl photocathode generated a photocurrent density increased by 40% compared to the bare Cu_2_O photocathode. Sun et al. constructed a hybrid TiO_2_ photoanode, with surface adsorbed by a Ru‐based molecular catalyst, together with a molecular photosensitizer, which displayed a photocurrent density of 70 μA cm^−2^ upon illumination without any external bias.^[59]^

It has been announced that a rational regulation of interfacial interaction mode between semiconductor and molecular complex, especially the type and density of anchoring group, could greatly enhance the PEC reaction activity via building efficient charge transfer channel, as well as improve the reaction stability through immobilization of molecular catalyst from desorption from electrode. Moreover, the linker between the anchor and molecular complex would also influence the charge transfer kinetics through its length, conjugation properties, and etc., as discussed in the following sections.

## 3 ANCHORING GROUP ENGINEERING BETWEEN SEMICONDUCTOR AND MOLECULAR COMPLEX

Covalent linkage is highly esteemed in interaction between semiconductor and molecular complex because it could provide an efficient charge transfer channel via a chemical bonding, and simultaneously inhibit attachment loss of molecular complex from semiconductor surface.^[60]^ For example, Wan et al. designed and established a P‐O‐M chemical bonding at the interface of BiVO_4_ and [Cp*Ir{P(O)(OH)_2_}_3_]Na.^[40]^ Subsequently, they adopted a strategy of oxidative transformation to remove Cp* and P(O)(OH)_2_ ligands and establish a stronger connection, that is, Ir‐O‐M bond formed between catalyst and oxide. Accordingly, the modified iridium‐BiVO_4_ hybrid photoanode showed 5.5 times enhancement of photocurrent intensity due to the catalyst anchored at surface overcoming the sluggish kinetics of BiVO_4_. For covalent linkage assembly, the key point is to develop suitable anchoring groups to link molecular complexes onto semiconductor surfaces for high performance. An excellent anchor should meet the demands of (i) tightly covalent connection with semiconductor surface, (ii) successful transfer of injected carriers, (iii) survive in aqueous environments, and (iv) bearing prolonged solar irradiation.^[61]^ In addition, the anchoring groups are asked to present shorter linkers and stronger electronic coupling to benefit electron injection for PEC water reduction, while longer linkers and weak electronic coupling are needed to hinder carrier recombination for the photoanode/molecular WOC system given the relatively long‐lived intermediate states formed accompanied by multiple electron transport.^[18]^

### 3.1 Introducing different anchoring groups for improved PEC water splitting performance

**FIGURE 2:**
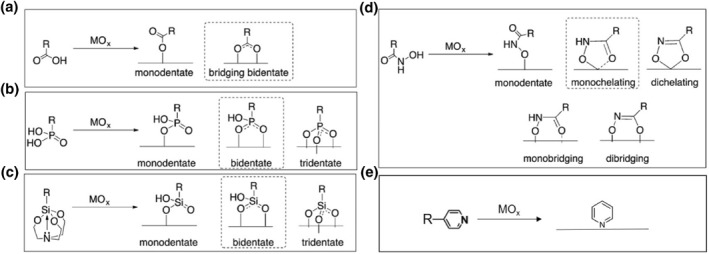
Possible binding modes of (a) carboxylic acid, (b) phosphonic acid, (c) silatranes, (d) hydroxamic acid, and (e) pyridyl anchors when bound to a metal oxide (MO_x_) surface. Reproduced with permission.^[61]^ Copyright 2017, Royal Society of Chemistry.

Carboxylic and phosphonic acids, as common anchoring groups, have been widely studied to covalently anchor molecular complexes onto surfaces of metal‐oxide‐based electrodes.[^60^, ^61^, ^62^, ^63^] The probable binding modes for carboxylic acids include monodentate and bridging bidentate (Figure 2a).^[61]^ Apart from monodentate and bidentate binding modes, the phosphonic acid has the possibility of a tridentate binding mode with metal oxide (Figure 2b). Considering that both carboxylic acids and phosphonic acids are suffering hydrolysis in aqueous environments, silatranes and hydroxamic acids, possessing more hydrolytically stability even in aqueous with wide pH values, have also been developed as anchoring groups. As shown in Figure 2c, the silatranes could be bound to metal oxide surfaces via Si‐O‐M bonds in different binding modes of monodentate, bidentate, and tridentate. Figure 2d describes the possible binding modes of the hydroxamic acid anchor when bound to a metal oxide surface, among which the monochelating mode is the most common one. The pyridyl anchors, preferentially forming coordinate bonds to metal ions (M‐N bonding) when exposed to metal oxide surface, have also been employed to link molecular complex and electrode for PEC water splitting (Figure 2e). The anchoring groups of silatranes and hydroxamic acids have been mainly employed in the dye‐sensitized PEC water splitting systems in the past few decades,[^64^, ^65^, ^66^, ^67^] while the carboxylic acids, phosphonic acids, and pyridyl anchors have been intensively adopted in the hybrid PEC photoelectrode for covalent bonding of molecular catalysts onto semiconductor surfaces.[^48^, ^60^, ^68^]

**FIGURE 3:**
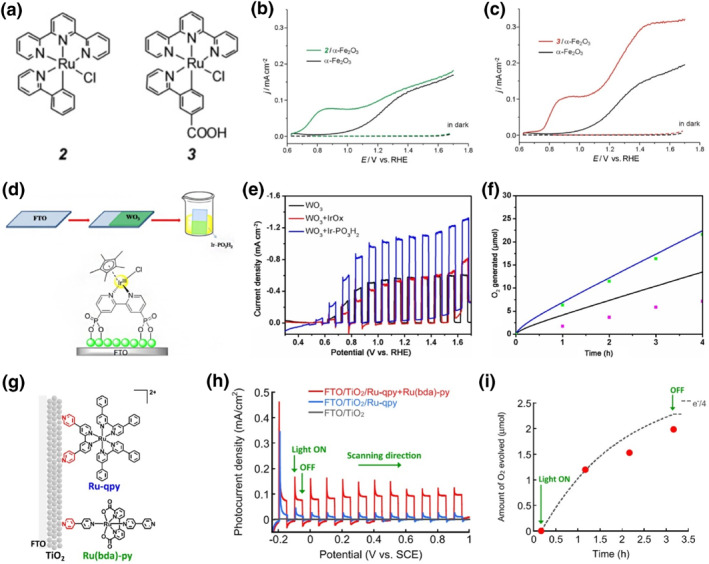
(a) Molecular structures of Ru(II) complexes. Linear voltammetry of (b) 2/α‐Fe_2_O_3_ and (c) 3/α‐Fe_2_O_3_ photoanodes in buffer solution (pH = 3) with or without illumination. Reproduced with permission.^[60]^ Copyright 2013, Elsevier. (d) Representations of proposed WOC (Ir‐PO_3_H_2_) molecular structure on a WO_3_ electrode surface. (e) Photocurrent‐potential characteristics under illumination for WO_3_, WO_3_+IrO_x_, and WO_3_+Ir‐PO_3_H_2_ photoanodes in KNO_3_ aqueous solution. (f) Photocatalytic oxygen‐evolution performance for the bare WO_3_ (pink and black) and WO_3_+Ir‐PO_3_H_2_ (green and blue) photoanodes in a KNO_3_ electrolyte under illumination at 1.23 V versus RHE. Reproduced with permission.^[48]^ Copyright 2017, Wiley. (g) The TiO_2_ hybrid photoanode for PEC water oxidation consisting of Ruqpy PS and Ru(bda) py WOC. (h) Linear sweep voltammetry curves of FTO/TiO_2_, FTO/TiO_2_/Ru‐qpy and FTO/TiO_2_/Ru‐qpy+Ru(bda)‐py photoanodes under intermittent solar light irradiation. (i) The amount of O_2_ evolved over time for the FTO/TiO_2_/Ru‐qpy+Ru(bda)‐py photoanode at the applied potential of 0.05 V *vs*. saturated calomel electrode (SCE). Reproduced with permission.^[28]^ Copyright 2023, American Chemical Society.

For the anchor of carboxylic acids, Chen et al. combined *α*‐Fe_2_O_3_ photoanode with a Ru(tpy)(ppy)Cl molecule without ‐COOH anchor group, and Ru(tpy)(pba)Cl with ‐COOH anchor group, respectively, for PEC water oxidation (Figure 3a).^[60]^ It has been demonstrated that with the surface attached by Ru(II) catalyst, the *α*‐Fe_2_O_3_ photoanode generated significantly enhanced photocurrent density and negatively shifted onset potential due to the high catalytic activities of Ru‐based molecular complexes (Figure 3b,c). More noteworthy is that as compared to *α*‐Fe_2_O_3_ with surface physically adsorbed by Ru(tpy)(ppy)Cl, the *α*‐Fe_2_O_3_/Ru(tpy)(pba)Cl structure with carboxylic linkage at interface displayed higher photocurrent density at high applied potential, as well as excellent stability without photocurrent density attenuation after 1000 s of PEC reaction. This encouraging result once again confirms that molecular complex engineered interface using a chemical bonding is quite beneficial for charge transfer and system stabilization. Similarly, the Cp*‐iridium water‐oxidation catalyst was reported to be attached to TiO_2_ surface via the carboxylic acid anchoring group for PEC water splitting by Moore et al.^[62]^ Correspondingly, a marked enhancement in PEC activity and stability was obtained with the TiO_2_/Cp*‐iridium hybrid photoanode. For phosphonic acid anchoring, Tong et al. synthesized a composite photoanode by anchoring a phosphate‐group‐modified iridium complex (Ir‐PO_3_H_2_) on WO_3_ through a covalent connection (Figure 3d).^[48]^ The obtained Ir‐PO_3_H_2_‐modified WO_3_ photoanode generated a photocurrent of 1.16 mA cm^−2^ at 1.23 V *vs*. RHE under simulated sunlight illumination, double that of the bare WO_3_ photoanode (Figure 3e). Moreover, the faradaic efficiency increased from 56% to 95% due to the accelerated surface charge transfer by the rapid surface kinetics of Ir‐PO_3_H_2_ (Figure 3f). Klepser et al. covalently anchored a phosphonate‐derivatized molecular complex, that is, Fe(tebppmcn)Cl_2_, onto a WO_3_ photoanode, dramatically increased the activity and selectivity of PEC water oxidation even in the absence of a sacrificial chemical oxidant due to the excellent stability of the Fe‐based molecular complex.^[68]^ Similarly, Rosser et al. immobilized a Ni(II) bis‐diphosphine (NiP) molecular catalyst onto TiO_2_ surface via a phosphate group for PEC water reduction.^[52]^ Simultaneously, they also linked the Fe(II)‐based molecular catalyst with WO_3_ via phosphate connection for water oxidation. Subsequently, the TiO_2_ hybrid cathode was coupled with the Fe‐catalyst modified WO_3_ photoanode to build a two‐electrode PEC configuration, which produced an onset of photocurrent located at approximately 0.6 V, much lower than the thermodynamic water splitting potential.

Given that both carboxylic and phosphonic acid groups suffer surface hydrolysis in aqueous environments, it is necessary to develop a stable anchoring group for tight binding of molecular complexes over semiconductor surfaces. Inspired by the “pyridyl anchoring technique” proposed by Ozawa et al., which enabled the polypyridyl ruthenium sensitizer closely attached onto TiO_2_ surface with stable Ti‐N bonding in dye‐sensitized PEC cell for water splitting in aqueous media,^[69]^ the strategy of pyridyl anchoring has also been intensively developed and employed in the fields of catalytic PEC cells in the past few decades.[^43^, ^70^, ^71^, ^72^] It has been illustrated that the pyridyl anchors could provide much stronger chemical linkages onto TiO_2_ surface than conventional carboxylate and phosphonate ones. Ozawa et al. linked TiO_2_ with both Ru‐based molecular photosensitizer and Ru‐based molecular WOC via pyridyl anchor (Figure 3g).^[28]^ PEC performance measurements showed that the molecular complex‐modified TiO_2_ photoanode produced an increased photocurrent density of ca. 90 μA cm^−2^ (Figure 3h), and a quantitative Faradaic efficiency (94 ± 6%) over 3 h under solar light irradiation (Figure 3i), confirming the effectiveness of “pyridyl anchoring technique” for improvement of the long‐term PEC stability of molecular complex‐modified photoanode used for water splitting. Luo et al. also employed a pyridyl anchor‐based molecular WOC (RuPy) in a TiO_2_‐based hybrid PEC system.^[72]^ The modified TiO_2_ photoanode could yield a steady photocurrent density of 42 μA cm^−2^ in 3600 s when applied in a two‐electrode system with 0 V bias voltage. It has been demonstrated that the introduction of pyridyl anchoring groups significantly improved the adsorption firmness on TiO_2_ in aqueous solution through Ti‐N bond. Recently Meyer et al. disclosed an important dependence for a surface‐bound catalyst on surface group.^[73]^ They found that a Ru‐bda catalyst with phosphonate binding could form surface‐bound μ‐oxo‐bridged dimers rapidly during the water oxidation process, thus greatly decreasing the reaction activities. On the contrary, this dimerization process would not occur by replacing the phosphonate acid binding group with a pyridine anchor. In addition to pyridyl anchoring, Wang et al. employed a hydroxamate anchor, displaying superior anchoring capability, to connect nickel and cobalt complexes with p‐Si/TiO_2_ for PEC water reduction, and obtained superior PEC activity and stability compared to previously reported molecular catalyst modified planner p‐Si photocathodes.^[21]^ Considering the complicated synthetic process required in introduction of carboxylic, phosphonic, or pyridyl binding groups, Liu et al. made full use of the four hydrophobic tert‐butyl groups in Co(salophen) complexes and bind them to BiVO_4_ surface with strong chemical linkage.^[74]^ In accordance, an extremely stable photocurrent of more than 3.5 mA cm^−2^ at 1.23 V *vs*. RHE was sustained for at least 3 h without decay. Therefore, it is important to strike a balance by considering the property and synthesis difficulty of the anchoring group.

### 3.2 Anchor group density affecting PEC water splitting properties

**FIGURE 4:**
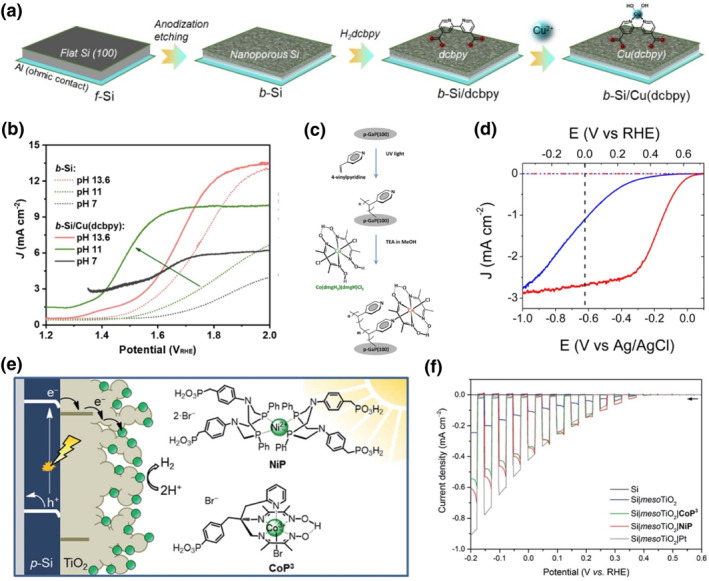
(a) Fabrication of b‐Si/Cu(dcbpy) photoanode from the flat Si (100) wafer. (b) J‐V curves of b‐Si/Cu(dcbpy) photoanode at pH 7.0, 11.0 and 13.6. Reproduced with permission.^[75]^ Copyright 2022, Wiley. (c) Schematic representation of the attachment method used to assemble modified photocathodes. (d) Linear sweep voltammetry curves of polyvinylpyridine‐grafted GaP (blue) and cobaloxime‐modified GaP (red) in the dark (dashed lines) and with 100 mW cm^−2^ illumination (solid lines). Reproduced with permission.^[77]^ Copyright 2013, American Chemical Society. (e) Schematic diagram of PEC H_2_ evolution with the Si|TiO_2_|catalyst photocathode and chemical structures of the immobilised catalysts NiP and CoP_3_. (f) Linear sweep voltammograms of Si (black), Si|TiO_2_ (blue), Si|TiO_2_|CoP_3_ (green), Si|TiO_2_|NiP (red) and Si|TiO_2_|Pt (grey) electrodes under chopped illumination. Reproduced with permission.^[81]^ Copyright 2017, Royal Society of Chemistry.

Anchoring group density has a positive correlation with molecular complex capture and immobilization onto semiconductor surface, leading to enhanced activity and stability for PEC water splitting. On the one hand, it is practicable to increase the surface active sites of semiconductors, especially for Si or In/Ga‐containing electrodes, to covalently grasp more molecular complexes via anchoring groups. For example, Jian et al. took an anodization etching method to treat the flat Si (f‐Si) and obtained the nanoporous structured Si (b‐Si), which enabled abundant Cu(dcbpy) catalysts with carboxylic acid anion groups chemisorbed on the Si surface (Figure 4a).^[75]^ In accordance, the prepared b‐Si/Cu(dcbpy) exhibited a significantly increased photocurrent density of 6.31 mA cm^−2^ at 1.5 V *vs*. RHE, much higher than that of the b‐Si photoanode (1.03 mA cm^−2^) (Figure 4b). Moore et al. put forward an approach of ultraviolet (UV)‐induced immobilization chemistry of alkenes to p‐GaP and p‐Si surfaces, which provides a means for patterning molecular linkers with attachment points to catalysts.[^76^, ^77^, ^78^, ^79^] Taking p‐GaP for example, the detailed attachment method used to assemble Co‐based complex WEC modified p‐GaP is presented in Figure 4c.^[77]^ Vinylpyridine was first photochemically grafted onto p‐GaP surface by UV illumination, followed by chemical treatment of the polyvinylpyridine (PVP) functionalized surface to anchor the Co‐based molecular catalyst via pyridine binding. The Co‐functionalized p‐GaP photocathode yielded a 2.4 mA cm^−2^ current density at a potential of 310 mV below the equilibrium potential of H^+^/H_2_, much higher than the PVP‐grafted p‐GaP photocathode (Figure 4d). Unlike that the unfunctionalized p‐GaP exhibited a 76% reduction in photocurrent after 5 min of irradiation, the cobaloxime‐modified cathode was relatively stable, with only a 17% decrease in photocurrent under the same conditions. Employing metal oxides as the interfacial layer linker between non‐oxide semiconductor and molecular complex is also a feasible choice to easily graft molecular complexes via covalent coordination. For instance, the Turner group deposited a TiO_2_ coating on GaInP_2_ surface to bind the metal complex of (HOOCpy)Co(dmgH)_2_(Cl) through a carboxylate covalent linkage.^[80]^ With successful attachment of the Co‐based molecular catalyst on GaInP_2_, the onset potential was positively shifted by about 650 mV compared to that of GaInP_2_/TiO_2_. With the same strategy, Reisner et al. deposited a mesoporous TiO_2_ scaffold layer onto p‐Si surface to anchor a Ni bis(diphosphine) catalyst for PEC water reduction (Figure 4e).^[81]^ A photocurrent onset potential of approximately 0.4 V versus RHE and an enhanced photocurrent density have been observed with the p‐Si/TiO_2_/NiP photocathode due to the well matched band energy between p‐Si and TiO_2_ and efficient catalytic properties of NiP (Figure 4f). Wu et al. introduced an Al_2_O_3_ thin layer to bind with more cobalt porphyrin complexes (CoTCPP) via carboxlate groups on BiVO_4_ for PEC water oxidation, giving rise to dramatically increased PEC activity and stability.^[82]^ Herein, it is necessary to consider that whether the surface treatment of semiconductor would introduce some defects as recombination centers inhibiting carrier efficient transport or not.

**FIGURE 5:**
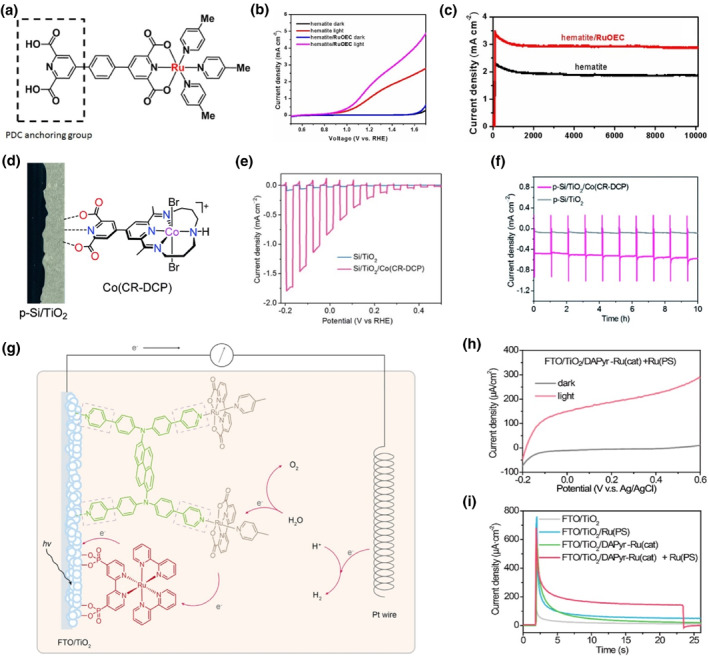
(a) The molecular structure of the Ru catalyst. (b) Linear sweep voltammetry curves of the bare hematite photoanode and catalyst Ru modified hematite photoanode in 1 M KOH solution under visible light illumination. (c) The photocurrent decay curves of bare hematite and hematite/Ru photoanodes in 1 M KOH solution under visible light illumination with an applied 1.4 V versus RHE bias. Reproduced with permission.^[84]^ Copyright 2015, Wiley. (d) Schematic illustration of the Si/TiO_2_/Co(CR‐DCP) hybrid photocathode. (e) Linear sweep voltammetry curves of Si/TiO_2_/Co(CR‐DCP) and bare Si/TiO_2_ in 0.1 M acetate buffer at pH 4.5 under chopped illumination. (f) Current‐time curves for Si/TiO_2_/Co(CR‐DCP) and Si/TiO_2_ at 0 V under illumination. Reproduced with permission.^[85]^ Copyright 2021, Royal Society of Chemistry. (g) Schematic representation of the hybrid PEC system for water splitting. (h) PEC linear sweep voltammetry scans from +0.6 to −0.2 V *vs*. Ag/AgCl conducted on the FTO/TiO_2_/DAPyr‐Ru(cat)+Ru(PS) electrode. (i) Photocurrent density generated on the FTO/TiO_2_/DAPyr‐Ru(cat)+Ru(PS) electrode and the control experiments in Na_2_SO_4_ solution. Reproduced with permission.^[86]^ Copyright 2024, American Chemical Society.

On the other hand, the design and synthesis of an anchoring group containing more covalent linkages, enabling more chemical bonds to form, is also feasible to build stronger connection between semiconductor and molecular complex. It has been demonstrated that the 2,6‐pyridinedicarboxylic acid (PDC), also named 2,6‐dicarboxypyridin‐4‐yl (DCP), with tridentate anchor, shows impressive stability when attached to metal oxide surface even submerged in aqueous solution.[^83^, ^84^, ^85^] For instance, Sun et al. immobilized a ruthenium molecular catalyst containing a strong PDC anchoring group (Figure 5a) on *α*‐Fe_2_O_3_ surface for PEC water oxidation, leading to improved photocurrent and high stability for over 10,000 s in a 1 M KOH solution. The enhanced PEC performance was due to the strong linkage between catalyst and *α*‐Fe_2_O_3_ (Figure 5b, c).^[84]^ Additionally, Wang et al. presented a Si/TiO_2_/Co(CR‐DCP) (cobalt tetraazamacrocyclic) hybrid structured photocathode, in which the Co(CR‐DCP) catalyst was chemically anchored with TiO_2_ via DCP bonding (Figure 5d).^[85]^ The immobilization of Co(CR‐DCP) boosted the photocurrent density of the hybrid p‐Si photocathode up to −682 μA cm^−2^ at 0 V *vs*. RHE, a factor of 31 compared to that of p‐Si/TiO_2_ (Figure 5e). Meanwhile, a steady photocurrent over 10 h of controlled potential photoelectrolysis experiment was also displayed with the hybrid photocathode (Figure 5f). Very recently, Zhong et al. coassembled a Ru complex photosensitizer and a Ru complex WOC on the TiO_2_ electrode, in which the Ru‐based WOC molecules were anchored onto TiO_2_ surface via metal‐pyridine coordination containing multiple pyridine groups (Figure 5g).^[86]^ An anodic photocurrent density of about 200 μA cm^−2^ at 0.2 V *vs*. Ag/AgCl was achieved with the hybrid TiO_2_ photoanode (Figure 5h). It should be simultaneously noted that the amount of covalent linkage in an anchor could affect the orientation of anchoring group toward semiconductor surface, which might influence the PEC activity and stability of the hybrid photoelectrode. On the whole, it has been well recognized that increasing the anchor group density reasonably could help to strengthen the semiconductor/molecular complex interfacial covalent interaction, thus enhancing the water splitting properties of the hybrid PEC system.

### 3.3 Anchoring groups affecting charge transfer kinetics

**FIGURE 6:**
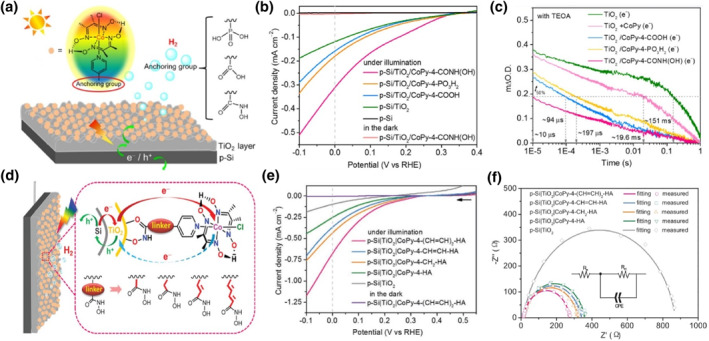
(a) Schematic illustration of the p‐Si/TiO_2_/CoPy‐4‐X hybrid photocathodes. (b) Linear scan voltammograms of the as‐prepared photocathodes and the reference electrodes under continuous illumination. (c) Transient absorption spectroscopy decays corresponding to the photoexcited electrons in the TiO_2_ layer for the as‐prepared FTO/TiO_2_/CoPy‐4‐X electrodes and bare FTO/TiO_2_ in the presence and absence of CoPy, measured at pH 9 with triethanolamine (TEOA, 0.1 M) as a hole scavenger. Reproduced with permission.^[88]^ Copyright 2019, American Chemical Society. (d) Schematic illustration of as‐prepared p‐Si|TiO_2_|CoPy‐4‐L‐HA photocathodes with different linkers. (e) Linear scan voltammograms of the as‐prepared photocathodes under continuous illumination. (f) Nyquist plots under illumination at 0 V for as‐prepared photocathodes and the reference electrode p‐Si|TiO_2_ (inset: Randles equivalent circuit diagram). Reproduced with permission.^[91]^ Copyright 2021 Wiley.

It has been illustrated that various anchors could also affect the electron‐hole transfer kinetics and charge recombination of the hybrid photoelectrodes for PEC H_2_ and O_2_ evolution.^[87]^ In order to clarify this point, Wang et al. constructed a hybrid photocathode, in which three cobaloxime complexes with different anchoring groups at the para position of the axial pyridine ligand were chemically attached to the TiO_2_ layer grown on p‐Si surface (Figure 6a).^[88]^ The as‐prepared p‐Si photocathode with an anchoring group of hydroxamate displayed the highest photocurrent density among the hybrid photocathodes (Figure 6b). Subsequently, Wang et al. studied the interfacial electron‐transfer dynamics from TiO_2_ layer to immobilized cobaloxime catalyst and carrier recombination via transient absorption spectroscopy (TAS). As shown in Figure 6c, the t_50%_ of photogenerated electrons for the electrode with hydroxamate anchor exhibited shorter value than that observed for the electrode with a carboxylate or a phosphonate, indicating that the effective electron transfer from TiO_2_ layer to the surface‐immobilized cobaloxime by hydroxamate anchor.

In addition to anchors, the linker between molecular catalyst and anchor could also make an impact on photocarrier separation and transport.^[87]^ On one way, the length of a linker could determine the spatial separation of a molecular catalyst from the semiconductor surface. Durrant et al. proposed that introduction of a linker enhancing the distance between semiconductor and catalytic core benefits to reduced electron‐hole recombination, which is crucial for water oxidation with slow multi‐electron catalytic reduction of protons. However, the long‐length linker might retard charge transfer from semiconductor surface to molecular catalyst simultaneously. On the other way, the conjugation property of the linker could also influence its conductive capability for electron transfer.[^89^, ^90^] In order to better understand the effect of different linkers on charge transfer kinetics of the hybrid photoelectrodes, Wang et al. immobilized the molecular cobaloxime catalysts bearing different linkers, that is, CoPy‐4‐L‐HA (CoPy = cobaloxime; L = linker, namely, none, CH_2_, CH=CH, and (CH=CH)_2_; HA = hydroxamate anchor), onto the p‐Si photocathode coated with TiO_2_ layer on surface (Figure 6d).^[91]^ The as‐prepared p‐Si hybrid photocathode with a linker of (CH=CH)_2_ displayed the highest PEC activity (Figure 6e), indicating that a better PEC performance could be acquired by extending the conjugation chain of the linker despite increasing the linker length at the same time. It was illustrated that the p‐Si|TiO_2_|CoPy‐4‐(CH=CH)_2_‐HA electrode displayed the smallest electron transfer resistance (Figure 6f), which could be further verified by the TAS test result that the longest t_50%_ for photogenerated holes with the highest initial amplitude was obtained with the FTO|TiO_2_|CoPy‐4‐(CH=CH)_2_‐HA electrode due to the most efficient suppression of surface charge recombination. Moreover, the flexibility of linkers is capable of affecting the ease of molecular catalyst synthesis for desired redox reaction.^[92]^ In order to further disclose the impact of anchoring groups on charge transfer dynamics, and rationally design suitable anchoring groups favoring carrier transport, more advanced characterizations are needed in addition to TAS.

## 4 CONCLUSIONS AND PERSPECTIVE

Chemical immobilization of molecular catalysts on semiconductor photoelectrodes via rational anchoring groups is of great significance to improve the water splitting performance of the hybrid PEC system. In recent years, with the development of molecular complex preparation and detection method technologies, more regulation strategies on anchoring groups have been reported successively in order to favor charge transfer from electrode surface to covalently immobilized molecular catalyst for outstanding PEC performances. In this mini review, we have timely summarized the recent advancements in research on anchoring group engineering techniques for efficient hybrid PEC water splitting. Introduction of an anchor group is realistic to build chemical bonding between a molecular catalyst and semiconductor photoelectrode favoring carriers transport and stabilization of the hybrid structure. The anchoring groups (e.g. pyridyl anchor, hydroxamate anchor, and etc.), displaying more hydrolytically stability than conventional anchors such as carboxylic acids and phosphonic acids, can provide much stronger chemical linkages onto the photoelectrode surface for PEC water splitting. Increasing anchoring group density gives great enhancement to PEC water splitting performances by capturing and immobilizing more molecular catalysts onto semiconductor surfaces. In addition, the anchor types, the linker length, and the conjugation property of linker could also influence the carrier transfer kinetics for PEC H_2_ and O_2_ evolution. Despite significant progress has been made in constructing hybrid molecular catalysts‐modified photoelectrodes with high‐efficiency PEC activities and outstanding stabilities, there is still a significant gap between the reported hybrid PEC systems and their wide applications. In the following aspects, we try to suggest possible directions of efforts to achieve this goal.

In order to further improve the PEC activity and stability of the hybrid PEC system, it is important to combine the different anchoring group regulation strategies. For example, it is feasible to choose the more hydrolytically stable anchor and equip it with the linker showing suitable length and conjugation property to enable efficient carrier transport during long‐term PEC operation for the hybrid system. Given the currently commonly used anchors are still facing the challenge of hydrolysis in aqueous environments, efforts should be continuously paid on developing new anchoring groups with long lifetime for tight binding during PEC water splitting process, as well as equipping the anchors with suitable linkers in favor of charge transfer. Certainly, it is necessary to take both synthesis complexity and cost into consideration. At present, there are still seldom reports on the impact of anchoring groups on the charge transfer and performance of the semiconductor/molecular complex hybrid photoelectrodes. Therefore, much deeper investigations are needed to study the effects of the type, number and orientation of surface anchoring groups, as well as the length, conjugation property, flexibility and conductivity of linkers, on electron‐hole separation and charge transfer kinetics for PEC water reduction and oxidation, respectively. Simultaneously, it is vital to assemble the beneficial anchoring group to optimize the semiconductor/anchoring group/molecular complex system interfacial interaction for efficient hole or electron transport. In order to immobilize the molecular complex onto the semiconductor surface better, the anchoring group can be protected by an overlayer (e.g. TiO_2_, Al_2_O_3_, etc.) for further stabilization of PEC reaction.[^93^, ^94^] The anchoring group regulation could be coupled with other modification strategies such as structural modulation of molecular catalyst, electrolyte engineering, etc. to give much more enhancement on the water splitting performances of the hybrid PEC system.

Until now, the interfacial charge transfer dynamics and surface water redox kinetics of the semiconductor/molecular complex photoelectrode are still unclear. Advanced characterization techniques, such as TAS, Raman spectroscopy, X‐ray absorption spectroscopy (XAS), and their in situ or operando techniques as well as density functional theory (DFT) calculations should be combined to disclose the effect of anchoring group regulation on interfacial charge transfer dynamics, surface water redox reaction processes and pathways in the hybrid system assembly, and to study the invalidation mechanisms of the hybrid system during long‐term PEC water splitting operation.

It is practicable to combine the molecular catalyst‐modified hybrid photoelectrode with the photovoltaic device to achieve bias‐unassisted PEC water splitting in the future. In order to further increase the value of the hybrid PEC system, it is promising to couple the water reduction reaction with some high added‐value reactions such as sulfur oxidation reaction (SOR),^[95]^ or to couple the water oxidation reaction with CO_2_ reduction, nitrogen fixation, etc.[^96^, ^97^]

## CONFLICT OF INTEREST STATEMENT

The authors declare no conflicts of interest.
